# Factors associated with the composition of the gut microbiome in patients with established rheumatoid arthritis and its value for predicting treatment responses

**DOI:** 10.1186/s13075-023-03013-x

**Published:** 2023-03-02

**Authors:** Jung Hee Koh, Eun Ha Lee, Kwang Hyun Cha, Cheol-Ho Pan, Donghyun Kim, Wan-Uk Kim

**Affiliations:** 1 Division of Rheumatology, Department of Internal Medicine, School of Medicine, The Catholic University of Korea, Seoul, 06591 Republic of Korea; 2 Center for Integrative Rheumatoid Transcriptomics and Dynamics, School of Medicine, The Catholic University of Korea, Seoul, 06591 Republic of Korea; 3 Natural Product Informatics Research Center, KIST Gangneung Institute of Natural Products, Gangneung, 25451 Republic of Korea; 4 Department of Biomedical Sciences, Seoul National University College of Medicine, Seoul, Republic of Korea; 5 Department of Microbiology and Immunology, Seoul National University College of Medicine, Seoul, Republic of Korea; 6 Institute of Endemic Diseases, Seoul National University Medical Research Center, Seoul, Republic of Korea

**Keywords:** Microbiota, Rheumatoid arthritis, Anti-rheumatic agents, Treatment response, Age

## Abstract

**Background:**

We aimed to investigate the gut microbiota of patients with established rheumatoid arthritis (RA) who have been managed with disease-modifying anti-rheumatic drugs (DMARDs) for a long time. We focused on factors that might affect composition of the gut microbiota. Furthermore, we investigated whether gut microbiota composition predicts future clinical responses to conventional synthetic DMARDs (csDMARDs) in patients with an insufficient response to initial therapy.

**Methods:**

We recruited 94 patients with RA and 30 healthy participants. Fecal gut microbiome was analyzed by 16S rRNA amplificon sequencing; the resulting raw reads were processed based on QIIME2. Calypso online software was used for data visualization and to compare microbial composition between groups. For RA patients with moderate-to-high disease activity, treatment was changed after stool collection, and responses were observed 6 months later.

**Results:**

The composition of the gut microbiota in patients with established RA was different from that of healthy participants. Young RA patients (< 45 years) had reduced richness, evenness, and distinct gut microbial compositions when compared with older RA patients and healthy individuals. Disease activity and rheumatoid factor levels were not associated with microbiome composition. Overall, biological DMARDs and csDMARDs, except sulfasalazine and TNF inhibitors, respectively, were not associated with the gut microbial composition in patients with established RA. However, the combination of *Subdoligranulum* and *Fusicatenibacter* genera was associated with a future good response to second-line csDMARDs in patients who showed an insufficient response to first-line csDMARDs.

**Conclusion:**

Gut microbial composition in patients with established RA is different from that in healthy individuals. Thus, the gut microbiome has the potential to predict responses of some RA patients to csDMARDs.

**Supplementary Information:**

The online version contains supplementary material available at 10.1186/s13075-023-03013-x.

## Introduction

Rheumatoid arthritis (RA), one of the most prevalent chronic systemic autoimmune diseases, is characterized by inflammation of the synovium, as well as damage to cartilage and bone [1]. The pathogenesis of RA is attributed to complex interactions between genetic and environmental factors [2, 3]. For example, allelic variants of *HLA-DRB1* are the strongest genetic risk factor for RA development, followed by multiple variants of immunoregulatory genes [4]. Moreover, cigarette smoking, silicon, and exposure to textile dust are classified as external environmental risk factors for RA, while the microbiota is a representative internal environmental factor [3].

Several studies show that patients in the early and preclinical stages of RA display alterations in the gut microbiota, such as enrichment of the genus *Prevotella* [5–9]. Moreover, the importance of the gut microbiota in the development of RA was confirmed in various animal experiments. For example, germ-free mice lack differentiation of pro-inflammatory Th17 cells, which are implicated in autoimmune pathogenesis [10], and depletion of the intestinal microbiota by antibiotics or germ-free conditions prevent the development of RA in mice [11, 12]. In particular, *Prevotella copri*, which is found in new-onset patients, triggers arthritis in humanized SKG mice by increasing the number of Th17 cells and Th17-related cytokines [7]. Also, an oral pathogen, *Porphyromonas gingivalis*, is a possible etiological agent [13, 14]. The prevalence of periodontitis and *P. gingivalis* is higher in anti-citrullinated protein antibody (ACPA)-positive at-risk individuals without clinical arthritis [15]. *P. gingivalis* increased the production of Th17 cells and aggravated arthritic symptoms, including joint destruction, in murine model [14, 16]. The peptidylarginine deiminase (PPAD) enzyme of *P. gingivalis* is suggested as the underlying mechanism due to its citrullination activity and subsequent induction of ACPA [14]. Moreover, the periodontal microbiota is associated with the level of rheumatoid factor which affect humoral immune responses [17]. Recently, a virulence toxin, Leukotoxin A, of *Aggregatibacter actinomycetemcomitans* was shown to induce dysregulation of human PAD enzymes, thereby increasing endogenous citrullination [18]. Thus, as mentioned above, the gut microbiota is a possible candidate responsible for priming aberrant systemic immune responses in RA.

Treatment with disease-modifying anti-rheumatic drugs (DMARDs) such as methotrexate (MTX) affects the composition of the gut microbiota in both mice and humans, resulting in partial restoration of a healthy gut microbiome [19–21]. In addition, the gut microbiota, which includes orthologs related to purine and MTX metabolism, is associated with future clinical responses to MTX in patients with new-onset RA [22].

Most existing human microbiome studies have identified microbial alterations between treatment-naive new-onset or preclinical RA patients and healthy people [6, 8, 23–25]. These studies are useful in that they demonstrate a relationship between the microbiota and RA onset. Because there are complex interactions between long-term administration of DMARDs and gut microbial composition, gut microbiome studies in patients with established RA, who have been treated with DMARDs for a long time, are rare. Moreover, no studies have examined whether the composition of the gut microbiota predicts responses to second-line conventional synthetic DMARDs (csDMARDs: MTX, leflunomide, sulfasalazine, and hydroxychloroquine) after an insufficient response to initial csDMARD treatment in patients with established RA. In addition, the effect of biological DMARDs (bDMARDs: tumor necrosis factor inhibitors, interleukin (IL)-6 receptor antagonist, and T cell costimulatory inhibitor), bringing a paradigm shift in the management of RA, on the gut microbiota is understudied [1].

Herein, we characterized the gut microbiota profile of patients with established RA who had received DMARDs for a long time and investigated the correlation between gut microbial composition and clinical parameters. In particular, we examined whether bDMARDs administered via the parenteral route were associated with gut microbiota composition, and whether gut microbes predict clinical response to DMARD treatment in RA patients with an insufficient response to initial therapy.

## Methods

### Participants’ information and stool collection

Two groups of participants were recruited: (1) patients with RA and (2) a control group without known comorbidities, including autoimmune diseases. Patients with RA who fulfilled the 2010 ACR/EULAR classification criteria [26] were recruited from Seoul St. Mary’s Hospital, Seoul, Republic of Korea. Patients treated with DMARDs for at least a year were enrolled in this study. As a control, healthy participants were recruited from the Wonju Severance Christian Hospital, Wonju, Gangwon-do. All participants were aged ≥ 20 years. A tofacitinib user and 4 patients who had ever been diagnosed with malignancy were excluded. Tofacitinib is a per-oral medication classified as a targeted synthetic DMARD, a class of drugs different from csDMARDs or biological DMARDs. In addition, malignancy can affect the gut microbiome regardless of RA. Participants taking antibiotics, having sporadic colitis within the previous 3 months, or with a known history of inflammatory bowel disease or systemic autoimmune disease (other than RA), were excluded. All patients with RA were investigated with respect to disease activity and concomitant medication(s) at the time of stool collection (Table 1). RA disease activity was categorized according to the DAS28 as follows: DAS28 ≤ 2.6 (remission); 2.6 < DAS28 ≤ 3.2 (low disease activity); 3.2 < DAS28 ≤ 5.1 (moderate disease activity); and DAS28 > 5.1 (high disease activity) [27].

**Table 1:** Participants’ characteristics

	RA (*n* = 94)	Healthy control (*n* = 30)	*P*
Age, years	57.4 ± 10.2	47.9 ± 3.5	< 0.001
Female, *n* (%)	87 (92.6)	30 (100)	0.194
BMI, kg/m^2^	22.7 ± 2.7	23.9 ± 3.0	0.064
Smoking, *n* (%)	2 (2.1)	3 (10.0)	0.091
Disease duration, years	8.7 ± 7.9	-	-
ACPA-positive, *n* (%)	75/89^†^ (84.3)	-	-
RF-positive, *n* (%)	71/93^†^ (76.3)	-	-
Medications			
csDMARDs, *n* (%)			
Methotrexate	53 (56.4)	-	
Leflunomide	39 (41.5)	-	
Sulfasalazine	14 (14.9)	-	
Hydroxychloroquine	39 (41.5)	-	
csDMARD combination	29 (30.9)		
Biological DMARDs, *n* (%)	39 (41.5)	-	
Oral glucocorticoids, *n* (%)	64 (68.1)	-	
NSAIDs, *n* (%)	51 (54.3)		
Disease activity			
DAS28	3.0 ± 1.5		
Remission (DAS28 < 2.6), *n* (%)	46 (48.9)		
Low disease activity (2.6 ≤ DAS28 ≤ 3.2), *n* (%)	7 (7.5)		
Moderate disease activity (3.2 < DAS28 ≤ 5.1), *n* (%)	31 (33.0)		
High disease activity (DAS28 > 5.1), *n* (%)	10 (10.6)		

^†^Among all RA participants, information about ACPA and RF was available for 89 and 93 patients, respectively

*ACPA*, Anti-citrullinated protein antibody; *BMI*, body mass index; *csDMARDs*, conventional synthetic disease-modifying anti-rheumatic drugs; *DAS28*, Disease Activity Score in 28 Joints; *NSAID*, non-steroidal anti-inflammatory drugs; *RF*, rheumatoid factor

**Fig. 1:**
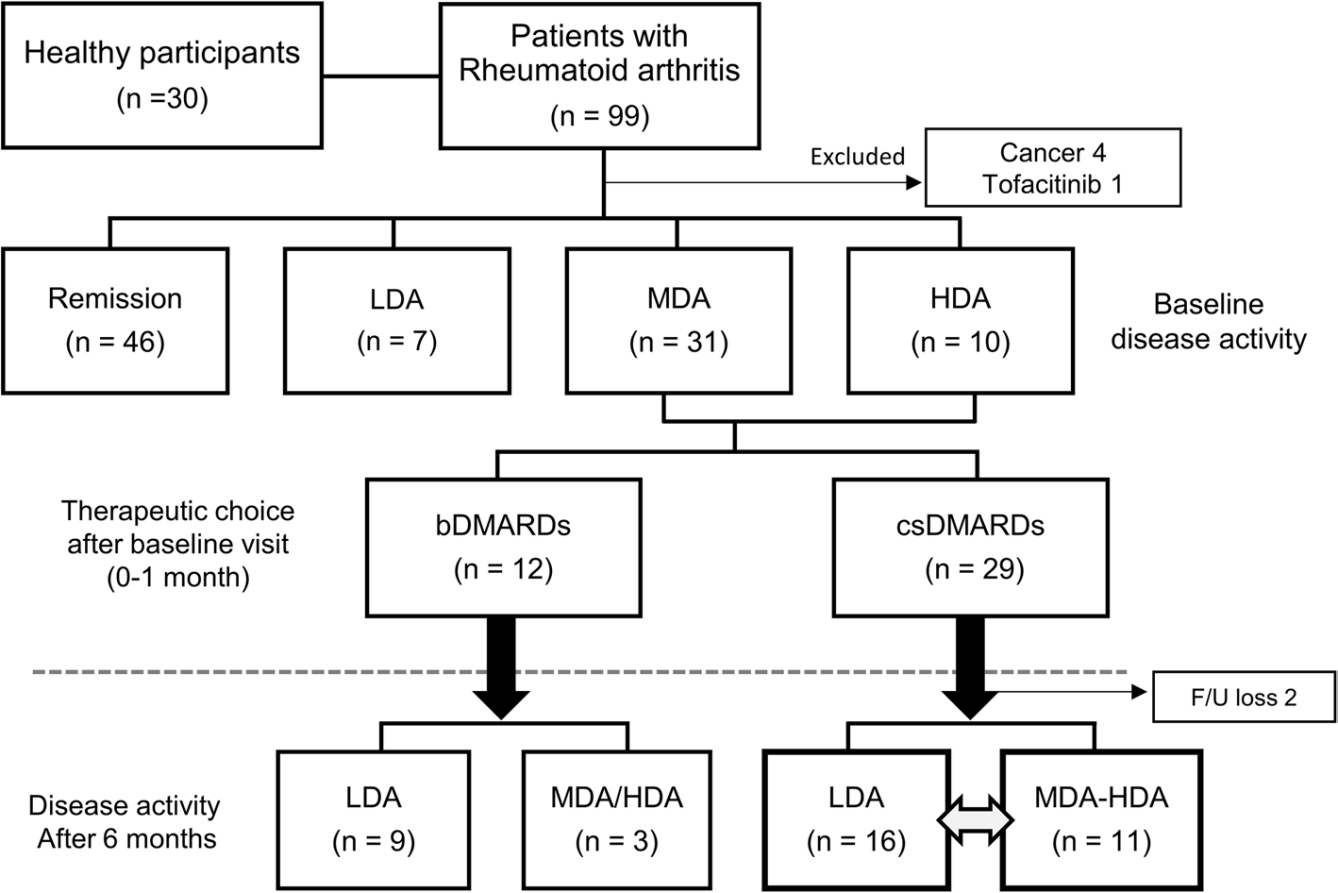
Flow chart showing the participants undergoing microbiome analysis. Overall, 99 patients with RA (who had been treated for at least a year) and 30 healthy participants were enrolled in the study. A tofacitinib user and 4 patients with malignancy were excluded from the final analysis. Patients with RA were divided according to the use of biological DMARDs, as well as disease activity (remission: DAS28 < 2.6, low disease activity: 2.6 ≤ DAS28 ≤ 3.2, moderate disease activity: 3.2 < DAS28 ≤ 5.1, high disease activity: DAS28 > 5.1), at the time of stool collection. In patients with moderate-to-high disease activity, therapeutic changes were recorded, and the response after 6 months was followed-up. bDMARDs, biological disease-modifying anti-rheumatic drugs; csDMARDs, conventional synthetic DMARDs; F/U, follow-up

Patients with moderate-to-high disease activity received modified treatment strategies at the discretion of the attending rheumatologist, and in agreement with the patient’s wishes. Changes in medication after stool collection and follow-up disease activity after 6 months were recorded to investigate whether the intestinal microbiota was associated with future treatment responses (Fig. 1). Treatment response was categorized as responder (DAS28 at 6 months’ follow-up ≤ 3.2) or non-responder (DAS28 at 6 months’ follow-up > 3.2) [27]. Stools were collected using disposable specimen containers (SPL Life Sciences, Pocheon, Korea) in accordance with a thorough step-by-step protocol. Samples were frozen immediately at − 20 °C and stored at − 80 °C until DNA extraction.

### Ethical approval

This study was approved by the institutional review board of Seoul St. Mary’s Hospital, the Catholic University of Korea (KC14TIMI0248), and Wonju Severance Christian Hospital (19–008). All study participants provided written informed consent. The study was following the principles of the Declaration of Helsinki. Informed consent was obtained from recruited patients before they participated in the study.

### DNA extraction, library preparation, and next-generation sequencing

DNA was extracted from fecal samples using the QIAamp PowerFecal Pro DNA Kit (Qiagen, Hilden, Germany). DNA was eluted with 50 μl of the elution buffer, and the concentration and purity were analyzed by agarose gel electrophoresis and Nanodrop 1000 spectrophotometry (Thermo Fisher Scientific, Wilmington, DE, USA), respectively. A 16S rRNA gene library was generated by PCR targeting the V3 to V4 regions [28]. Next-generation sequencing was conducted on the MiSeq system using a paired-end 2 × 250 bp platform (Illumina, San Diego, CA, USA). Generated raw data were deposited in the Short Read Archive database of NCBI (accession number PRJNA791216; https://www.ncbi.nlm.nih.gov/bioproject/PRJNA791216/).

### Microbiome bioinformatic analyses

The resulting raw reads were analyzed using QIIME2 (Release 2020.8) [29, 30]. Trimming and joining of paired-end reads were done using default parameters. Denoising to derive amplicon sequence variants (ASV) was carried out by DADA2. Taxonomic assignment to each ASV was conducted based on the SILVA database (Release 138). Data visualization and statistical analysis were performed using Calypso software (version 8.84) [31]. As α-Diversity, Chao1, Simpson, and Shannon indices were represented: Chao1 index estimates the numbers of observed species (richness), and Simpson and Shannon indices are an estimator for both species richness and evenness. PCoA plot is a β-diversity representing the distances between the microbiome of samples in a low-dimensional space. RDA and CCA aim to find relationships between microbial composition and multiple explanatory variables. Differences between groups were assessed using the default method for *p*-value correction using the web-application Calypso (FDR < 0.05). Differences in the abundance of microbes between responders and non-responders were identified using LEfSe (linear discriminant analysis (LDA) effect size) analysis (https://huttenhower.sph.harvard.edu/galaxy/).

### Additional statistical analyses

Differences in descriptive and outcome variables between groups were tested using a *t*-test (for continuous variables with a normal distribution) and a chi-square test (for categorical variables). The area under the receiver-operator curves (AUC) for relative abundance was calculated and used to differentiate responders from non-responders. Statistical analyses were conducted using SAS version 9.4 (SAS Institute, Cary, NC, USA), and graphs were drawn using GraphPad Prism 8 (GraphPad Software, San Diego, CA, USA).

## Results

### Clinical characteristics of the patients

Fecal samples were collected from 99 patients with RA and from 30 healthy controls. Among the RA participants, four diagnosed with cancer after stool collection and one treated with tofacitinib were excluded from further analysis (this is because cancer can affect the gut microbiome regardless of RA, and tofacitinib is classified as a targeted synthetic DMARD). Thus, 94 RA patients and 30 healthy participants were enrolled in the microbiome study (Fig. 1). Patients with RA (mean age, 57.4 ± 10.2; female, 93%) were older than healthy participants (mean age, 47.9 ± 3.5; all female) (Table 1). There were no statistical differences between the groups regarding smoking rates and body mass index (BMI) (Table 1). RA patients were classified as follows: remission (48.9%), low disease activity (7.5%), moderate disease activity (33.0%), and high disease activity (10.6%) (Fig. 1 and Table 1). ACPA and RF, the most used serological markers for RA diagnosis [32], were detected in 84.3% and 76.3% of RA patients, respectively (Table 1). Because the median duration of RA was 7 years (interquartile range (IQR), 3–12.5 years), the RA patients in this study had been treated with DMARDs for several years (Table 1). Of those with RA, 41.5% were treated with a bDMARDs, and 84.6% of bDMARD users were in combination with csDMARDs (Table 1). The remaining 58.5% of patients had received csDMARDs, but were bDMARD-naïve.

### Comparison of gut microbiota between RA patients and healthy controls

**Fig. 2:**
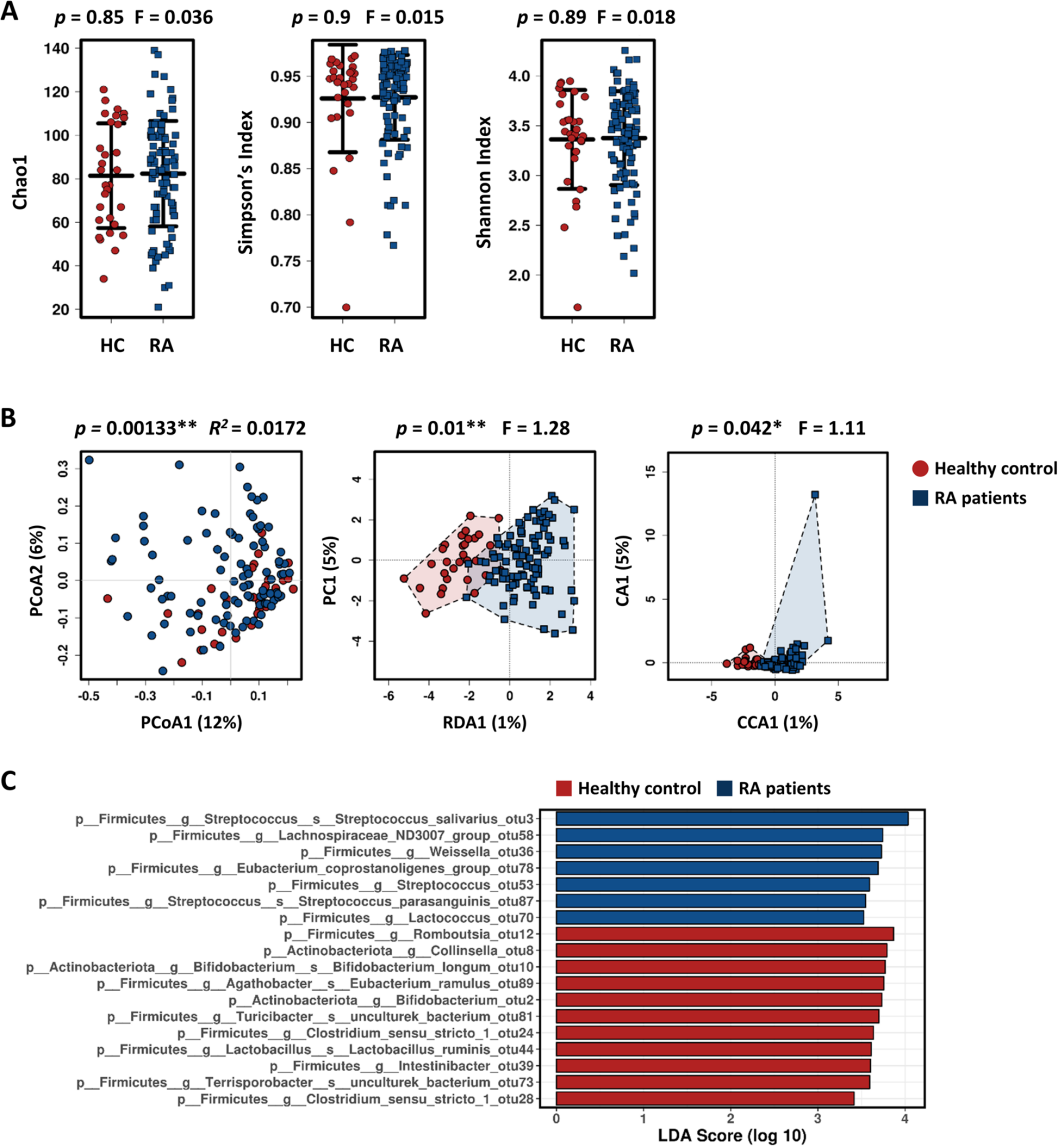
Comparison of the fecal microbiome between RA patients and healthy participants. **A** α-Diversity (shown as the Shannon index, Simpson’s index, and Chao1) based on Bray–Curtis operational taxonomic units (OTUs) data from healthy participants and RA patients. Bars show the mean ± SD. Each symbol represents individual participants. **B** Principal coordinate analysis (PCoA, left), redundancy analysis (RDA, middle), and canonical correspondence analysis (CCA, right) plots of the gut microbiota from RA patients and healthy participants at the OTU level. PC, principal component analysis. CA, correspondence analysis. **C** LEfSe revealed specific microbes at the OTU level

The MiSeq system provided 3,136,924 qualified sequences (median 23,913 reads per sample, range: 9020–67,198) of 16S rRNA amplicons from fecal samples of RA patients and healthy subjects. First, we compared gut microbial composition between RA patients and healthy subjects. The bacterial richness and diversity of RA patients were not different from those of healthy participants (Fig. 2A). However, principal coordinates analysis (PCoA) suggested a dissimilarity between RA patients and healthy controls (*p* = 0.00133, Adonis), although distinct clusters were not clearly observed in the plots (Fig. 2B). In line with β-diversity, redundancy analysis (RDA; *p* = 0.01) and canonical correspondence analysis (CCA; *p* = 0.042) suggested that RA had an impact on the gut microbiota (Fig. 2B). Additionally, we performed LEfSe analysis, which determines the features (the operational taxonomic units (OTU) level in this study) most likely to explain differences between groups. Genera *Streptococcus*, *Lachnospiraceae*, and *Weisselia* were relatively more abundant in patients with RA, whereas genera *Romboutsia*, *Collinsella*, *Bifidobacterium*, *Clostridium *sensu stricto* 1*, and *Lactobacillus* were enriched in healthy participants (Fig. 2C). These results indicate that, similar to previous studies of established RA patients [33, 34], the gut microbial composition of RA patients differs from that of healthy individuals.

**Fig. 3:**
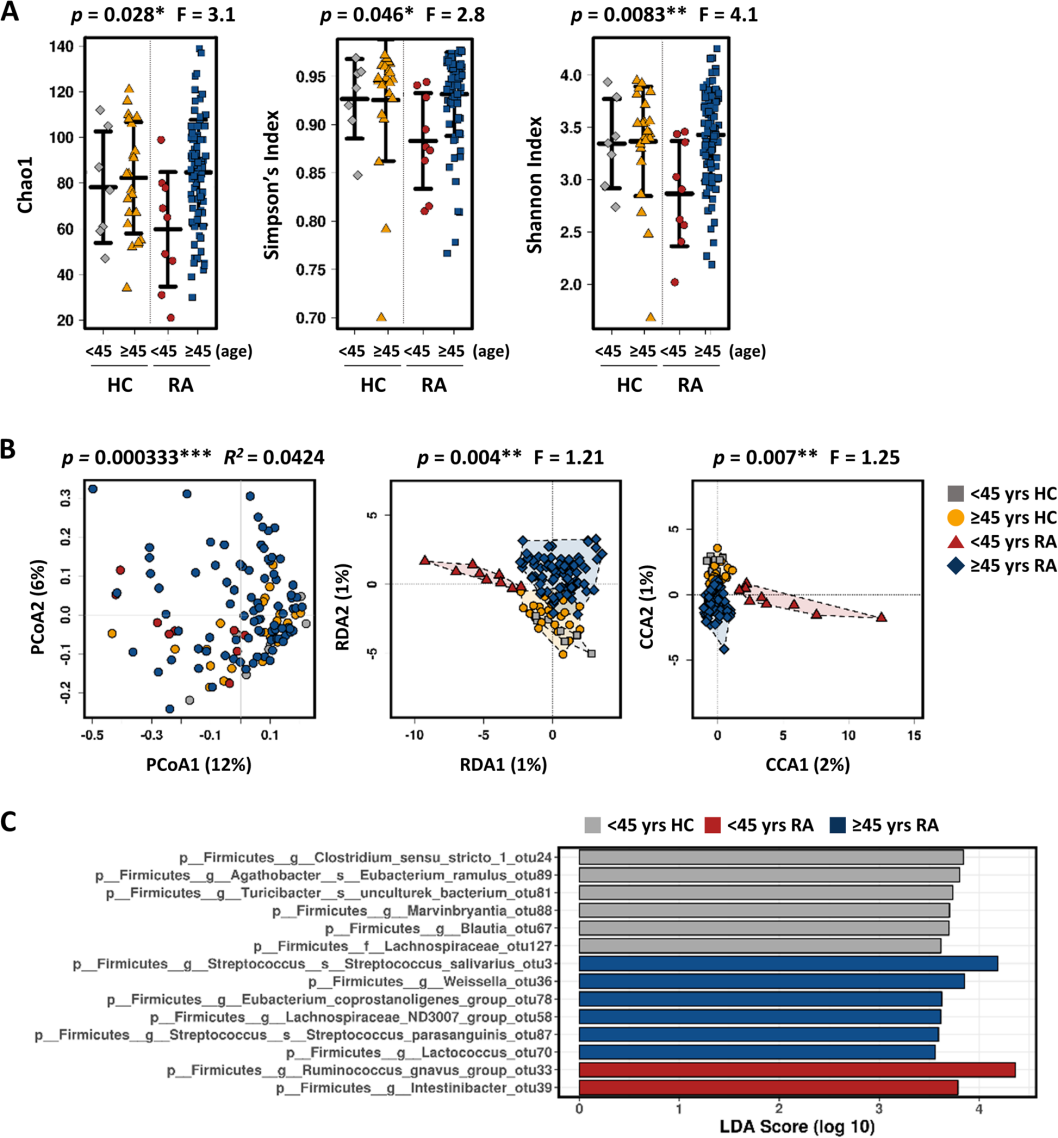
Correlation between age and gut microbiome composition in RA patients and healthy participants. Participants were divided into young (< 45 years) and senior (≥ 45 years). **A** α-Diversity (shown as Chao1, Shannon index, and Simpson’s index) based on Bray–Curtis OTUs data. **B** PCoA (left), RDA (middle), and CCA (right) plots at the OTU level. **C** LEfSe revealed specific microbes at the OTU level

When the gut microbiome was compared on the basis of participants’ characteristics, we found that young RA patients showed obvious discrepancies. The Chao1, Simpson, and Shannon indices showed that young RA patients (< 45 years) have significantly reduced microbial richness and diversity than older RA patients (≥ 45 years) (Fig. 3A). By contrast, such differences were not found in healthy participants (Fig. 3A). In addition, Bray–Curtis dissimilarity analysis revealed significant separations in the microbial community of young RA patients (Fig. 3B), which is supported by supervised RDA and CCA (Fig. 3B). In particular, young RA patients had an abundance of *Ruminococcus gnavus* and the genus *Intestinibacter* (Fig. 3C). By comparison, species *Streptococcus salivarius*, *Streptococcus parasanguinis*, genus *Weissella*, and the *Eubacterium coprostanoligenes*, *Lachnospiraceae* ND3007 group, and genus *Lactococcus* were enriched in older RA patients (Fig. 3C). These data suggest that the gut microbial composition plays an important role in RA pathogenesis, at least in young people.

### Composition of the gut microbiota according to RA disease activity

**Fig. 4:**
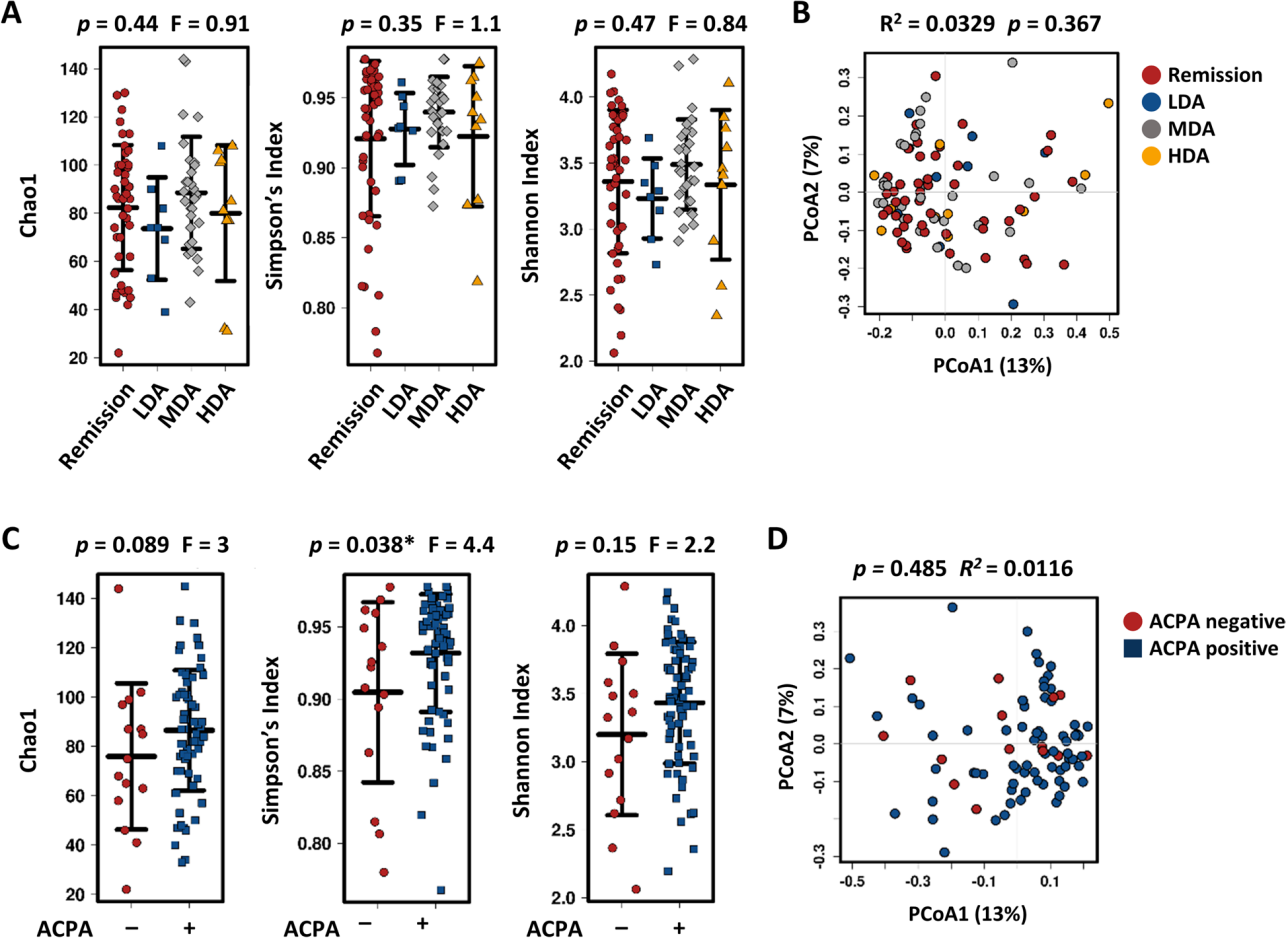
Effect of disease activity and ACPA on the gut microbiome in patients with RA. **A**, **B** Comparison of gut microbiome composition according to disease activity at the time of stool collection. Remission (DAS28 ≤ 2.6), low disease activity (LDA: 2.6 < DAS28 ≤ 3.2), moderate disease activity (MDA: 3.2 < DAS28 ≤ 5.1), and high disease activity (HDA: DAS28 > 5.1). α-Diversity (shown as the Chao1, Shannon index, and Simpson’s index) (**A**) and PCoA plot at the OTU level (**B**). **C**, **D** Comparison of gut microbiome composition between ACPA-positive and ACPA-negative RA patients. α-Diversity (shown as Chao1, Shannon index, and Simpson’s index) (**C**) and PCoA plots at the OTU level (**D**)

Next, we examined whether the gut microbial composition is associated with RA disease activity. When comparing the gut microbiome between groups classified according to disease activity, we found no differences in the α- and β-diversities (Fig. 4A, B). Additionally, we analyzed the gut microbial composition according to the presence or absence of ACPA and RF. Although the Simpson index in ACPA-positive RA patients was higher than that in ACPA-negative RA patients, there were no statistical differences in the Chao1 and Shannon indices or the β-diversity PCoA plot (Fig. 4C, D). Likewise, RF had no effect on the gut microbial community of RA patients (data not shown). These results indicate that there is no relationship between the gut microbial community and disease activity in established RA patients.

### Effect of medications on the gut microbiota

**Fig. 5:**
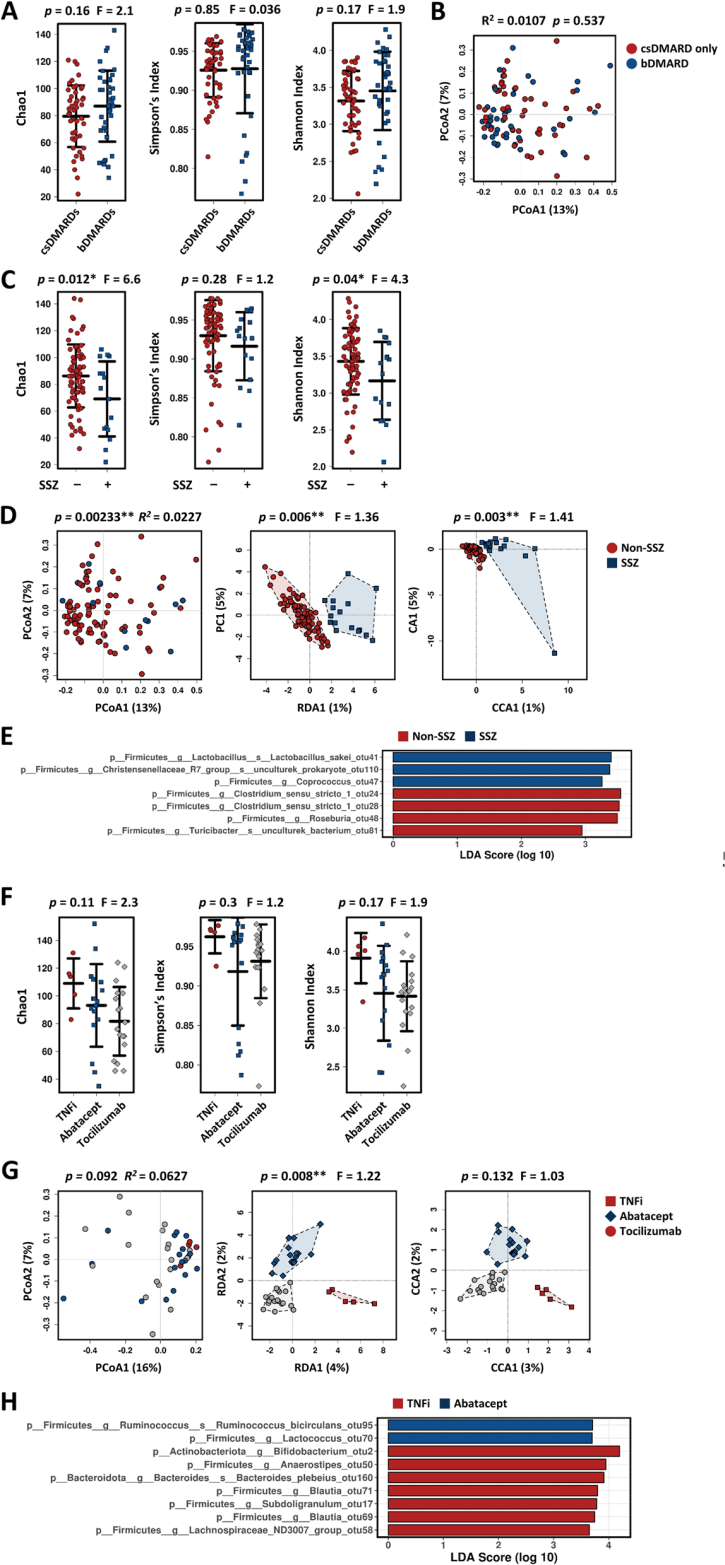
Comparison of gut microbiome composition according to the use of DMARDs. **A**, **B** Composition of the gut microbiome from RA patients treated with or without bDMARDs. α-Diversity (shown as Chao1, Shannon index, and Simpson’s index) (**A**). PCoA plot at the OTU level (**B**). **C–E** Comparison of gut microbiome according to the administration of sulfasalazine (SSZ). α-Diversity (shown as Chao1, Shannon index, and Simpson’s index) based on Bray–Curtis OTUs data (**C**). PCoA (left), RDA (middle), and CCA (right) plots at the OTU level (**D**). CA, correspondence analysis; PC, principal component analysis. LEfSe revealed the altered microbes at the OTU level (**E**). **F–H** Comparison of the gut microbiome according to bDMARD use (tumor necrosis factor inhibitors, (TNFi), abatacept, and tocilizumab). α-Diversity (shown as Chao1, Shannon index, and Simpson’s index) based on Bray–Curtis OTUs data (**F**). PCoA (left), RDA (middle), and CCA (right) plots at the OTU level (**G**). LEfSe revealed the altered microbes at the OTU level (**H**)

The gut microbiome is affected by external factors, such as dietary habits and per oral medications [35]. Because the RA patients in this study were diagnosed and treated with DMARDs for 1 year or more, their medications might influence the gut microbial community. When comparing the gut microbiome between patients treated with and without bDMARDs, we found no meaningful discrimination (Fig. 5A, B). Moreover, combined treatment with bDMARDs and csDMARDs had no meaningful impact on the gut microbial community (Supplementary Fig. 1A and 1B).

By extension, the gut microbiome was analyzed according to each kind of csDMARD and bDMARD. MTX was the most commonly prescribed csDMARD (62% of non-bDMARD users and 49% of bDMARD users), followed by leflunomide (47% of non-bDMARD users and 33% of bDMARD users), hydroxychloroquine (56% of non-bDMARD users and 21% of bDMARD users), and sulfasalazine (24% of non-bDMARD users and 3% of bDMARD users). MTX, leflunomide, and hydroxychloroquine had no significant influence on gut microbial composition (Supplementary Fig. 2A and 2B, and data not shown). By contrast, sulfasalazine reduced gut bacterial richness and evenness and altered the bacterial composition (Fig. 5C, D). Additionally, LEfSe analysis revealed that sulfasalazine increased *Lactobacillus sakei* species and *Christensenellaceae* R7 group and *Coprococcus* genera, while reducing *Clostridium* sensu stricto 1, *Roseburia*, and *Turicibacters* genera (Fig. 5E).

The bDMARDs used in this study were categorized according to their mechanism of action: tumor necrosis factor α (TNF-α) inhibitors (TNFi), IL-6 receptor inhibitors (tocilizumab), and cytotoxic T lymphocyte-associated protein 4 (CTLA-4) fusion protein (abatacept). None of these bDMARDs led to a significant alteration in the gut microbiome (Fig. 5F, G). However, TNFi treatment increased bacterial diversity and changed the microbial composition when compared with abatacept- or tocilizumab-treated subjects, albeit not significantly (Fig. 5F, G). The abundance of genera *Bifidobacterium*, *Anaerostipes*, *Bacteroides, Blautia*, *Subdoligranulum*, the *Lachnospiraceae* ND3007 group, and species *Bacteroides plebeius* were enriched in TNFi-treated patients (Fig. 5H). These results indicate that some drugs can affect the gut microbiota in established RA patients, regardless of administration route.

### The gut microbiota as a predictor of response to DMARDs

To identify gut bacteria that can predict treatment outcomes, we classified patients with active disease status (DAS28 ≥ 3.2 at the time of stool collection) as responders (DAS28 < 3.2 at 6-month follow-up) or non-responders (DAS28 ≥ 3.2) (Fig. 1). When comparing changes in the gut microbiome of responders and non-responders to all modified treatment strategies, the two groups showed no significant differences with respect to diversity and dissimilarity (Supplementary Fig. 3A and 3B). Non-responders had more genera *Lachnospiraceae* NK4A136 group and *Adelercreutzia* than responders, whereas no bacteria were significantly predominant in responders (Supplementary Fig. 3C). When the AUC values for genera *Lachnospiraceae* NK4A136 group and *Adelercreutzia* were calculated, these two genera showed modest predictive capacity for differentiating good responders from non-responders to second-line therapy (AUC = 0.669 and 0.665, respectively) (Supplementary Fig. 3D).

**Fig. 6:**
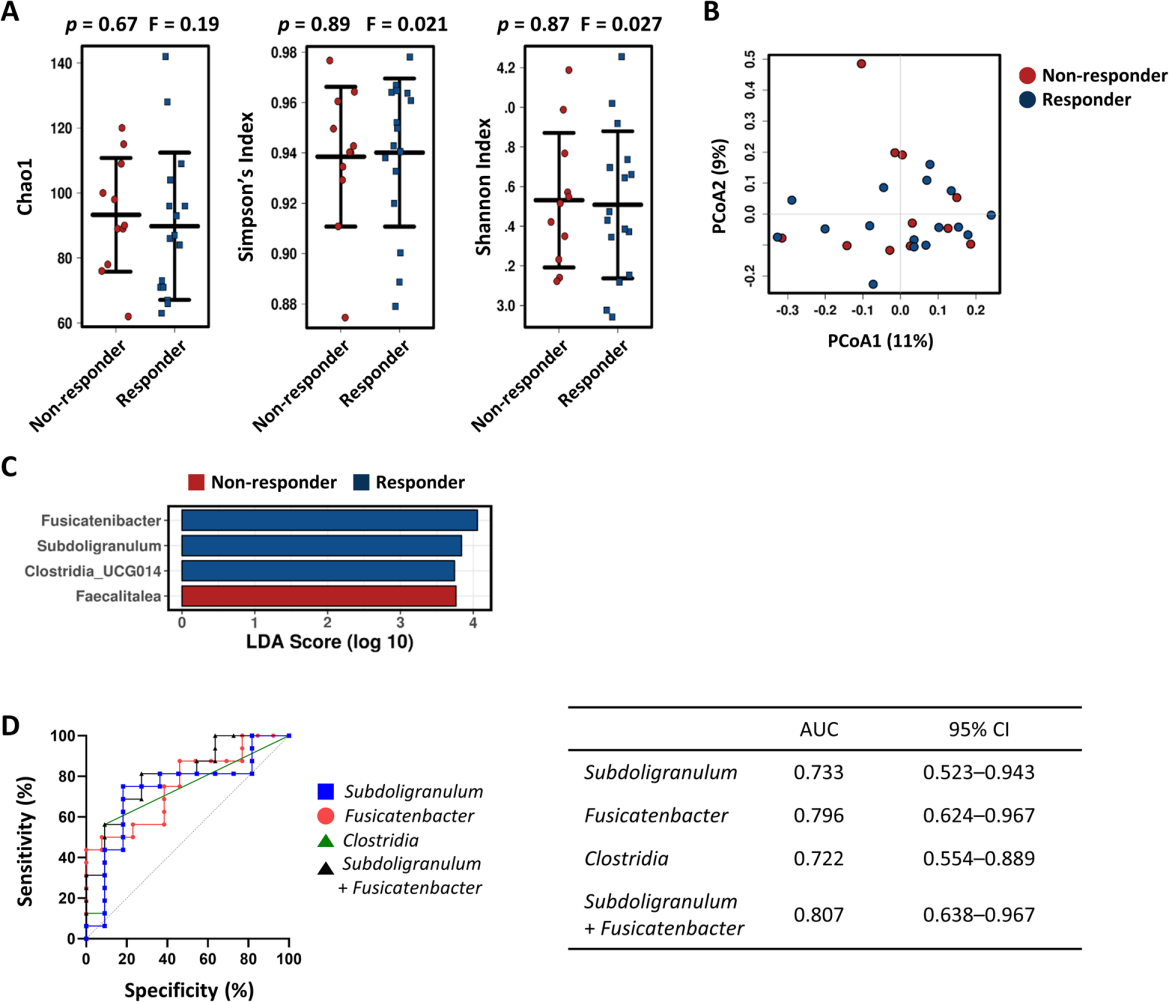
Comparison of the gut microbiome according to response to csDMARDs, and the potential of the gut microbiota to predict prognosis after the change. The baseline gut microbial diversity and taxa in RA patients with moderate-to-high disease activity according to the response to csDMARDs (after 6 months). Responders were defined as patients with ≤ 3.2 DAS28 at 6 months, and non-responders as those with > 3.2 DAS28 at 6 months. **A** α-Diversity (shown as Chao1, Shannon index, and Simpson’s index) based on Bray–Curtis OTUs data. **B** PCoA plot at the OTU level. **C** LEfSe revealed the altered microbes at the genus level. **D** The predictive potential of genera *Subdoligranulum*, *Fusicatenibacter*, and *Clostridia*, and a combination of genera *Subdoligranulum* and *Fusicatenibacter*, for predicting responses to csDMARDs. ROC curve for each genus, and combinations useful for predicting response to csDMARDs (left panel), along with the area under the curve (AUC) and 95% confidence intervals (right panel)

Next, we compared the gut microbiome of responders and non-responders treated with csDMARDs (Fig. 1). The number of patients who had glucocorticoid after the fecal collection was not different significantly between responders and non-responders in csDMARDs users (88% vs. 100%, respectively, *P* = 0.499). Although the α- and β-diversity were not significantly different (Fig. 6A, B), we found a relative expansion of genera *Fusicatenibacter*, *Subdoligranulum*, and *Clostridia* uncultured genera 014 in good responders, along with a contraction of *Faecalitalea* (Fig. 6C). Notably, the combination of *Fusicatenibacter* and *Subdoligranulum* distinguished good responders from non-responders well (AUC = 0.807) (Fig. 6D).

## Discussion

Since the beginning of this century, it has been known that the gut microbial composition of RA patients differs from that of healthy individuals [6, 7, 19, 34]. Here, analysis of β-diversity (PCoA) revealed perturbation of the microbial community in RA patients. However, some of our other results were inconsistent with previous reports [6, 7, 19, 34, 36]. For example, we observed no reduction in the richness and evenness of the RA-associated gut microbiome, nor any microbial alterations associated with RA disease activity. Most previous reports compared the gut microbiota of preclinical RA or new-onset RA patients with that of healthy individuals [6, 7, 19, 22, 34]. By contrast, we examined the gut microbiome of 94 RA patients treated with DMARDs for more than 1 year (established RA patients). Because RA is a chronic inflammatory disorder, our study of the gut microbiome in established RA patients has discriminative importance. However, microbiome analysis in established RA patients is more complex because drugs can alter gut microbial composition [20, 35, 37]. DMARD treatment partly restores a healthy gut microbiome in RA patients [19]. In other words, after treatment with DMARDs, the gut microbial composition of RA patients becomes more similar to that of healthy people. In addition, patients with RA have altered gut barrier integrity, and effective treatment restores gut permeability [38]. Considering the tight association between gut microbiota and gut epithelial integrity, restoring gut permeability may contribute to restoring gut microbiota. Thus, established RA patients may well have different gut microbiota from preclinical RA and new-onset RA patients, and the differences in the microbial community between RA patients and healthy participants may become less marked after RA treatment.

Consistent with a previous report, we found that some drug use alters the gut microbiota. Sulfasalazine is a prodrug that is converted to an active form by the intestinal microbiota via azoreductase [35]. The major anaerobic bacteria that produce azoreductase is the *Clostridium* and *Eubacterium* genera [39]. Some probiotics like *Lactobacillus acidophilus*, *Bifidobacterium lactis*, and *Streptococcus salivarius* are also capable of reducing azo compounds [40]. In the present study, *Lactobacillus sakei*, a probiotic, was increased in patients taking sulfasalazine, while the other azoreductase-producing microbe, *Clostridium *sensu stricto* 1*, was reduced. While sulfasalazine treatment regulated the abundance of microbes producing azo reductase, microbial alterations would affect the efficacy of sulfasalazine. Although the number of patients treated with TNFi was low, they had a distinct microbiota composition when compared to those treated with abatacept or tocilizumab. Patients treated with TNFi had a higher fecal abundance of the putative short-chain fatty acid (SCFA)-producing genera *Lachnospiraceae ND3007 group*, *Anaerostipes*, and *Subdoligranulum* [41–43]. Because SCFAs such as butyrate have beneficial effects on RA [44], microbial alterations might contribute to the therapeutic effects of TNFi.

One intriguing finding was that the gut microbial composition of young RA patients is clearly different from that of older patients and healthy controls with respect to α- and β-diversity. In addition, an abundance of *R. gnavus* was observed in the microbiota of young RA patients. *R. gnavus*, a strict anaerobic Gram-positive coccus, is a prevalent commensal found in the intestines of nearly 90% of people [44, 45]. Because *R. gnavus* uses sialic acid from mucin glycan layers as a carbon source, it resides mainly on the gut mucosal surface [45]. *R. gnavus* also produces an inflammatory polysaccharide and toxic metabolites that might contribute to local inflammation [46]. This result suggests that the gut microbiota may have a more important role in RA pathogenesis in particular age groups. Whether the abundance of *R. gnavus* contributes to RA prevalence in the young requires further study.

It is always challenging to modify treatment of RA patients who show an insufficient response to initial csDMARDs therapy because csDMARDs are relatively cheap and convenient (per oral medicine); however, bDMARDs often appear to be more effective (but are relatively more expensive) [47]. So far, there are no biomarkers that predict the effectiveness of second-line csDMARDs therapy. In this study, we asked whether particular gut microbe(s) could be used as a prognostic marker for predicting therapeutic responses. Among csDMARD-treated patients, those with an abundance of genera *Fusicatenibacter* and *Subdoligranulum*, and the order *Clostridia* UCG-014, showed favorable responses to csDMARDs therapy, whereas those harboring genus *Faecalitalea* were not. *Fusicatenibacter* and *Subdoligranulum* were depleted in RA patients but more abundant in healthy individuals [48, 49]. *Fusicatenibacter saccharivorans* levels correlate positively with the production of SCFAs, which effectively inhibit progression to arthritis [50, 51]. Also, *Fusicatenibacter saccharivorans* induce anti-inflammatory cytokine production by lamina propria mononuclear cells [52]. *Subdoligranulum* are negatively associated with inflammatory cytokines and acute phase reactants [41]. Moreover, our results indicate that the combination of *Fusicatenibacter* and *Subdoligranulum* predicts a favorable response to csDMARD treatment in patients who showed an insufficient response to initial DMARDs therapy. In other words, patients with a low abundance of *Fusicatenibacter* and *Subdoligranulum* may be better off switching to bDMARDs rather than continuing csDMARDs therapy (i.e., escalating the dose, changing, or adding on new csDMARDs). This suggests that gut microbes can predict whether csDMARDs therapy will still be effective in patients who did not respond to initial csDMARDs. Further studies are needed to confirm whether *Fusicatenibacter* and *Subdoligranulum* can be used as a prognostic biomarker for csDMARD treatment responses.

This study has several limitations. First, some microbes, which showed different abundance between groups, may be related to diet; however, we did not survey dietary habits. Second, this study did not analyze time-series samples from each patient; therefore, actual changes in microbiota cannot be tracked. Third, the number of patients treated with TNFi was not high enough to show statistical differences. Fourth, as we aimed to analyze the gut microbiome in established RA patients who were commonly encountered in clinical practice, we cannot completely exclude the confounding factors from various combinations of multiple drugs, including csDMARD combination. Therefore, it may not be suitable for examining the effect of a specific drug on microbial compositions. Nevertheless, the strength of this study is that it reveals correlations between gut microbiota composition and variables related to real-world patients with established RA. In addition, we show that microbial composition is a candidate biomarker for predicting treatment responses in patients treated with csDMARDs. Rheumatologists encounter a decision making to switch biological DMARDs or add or switch another csDMARDs when the treatment response to the current csDMARDs therapy is insufficient. Since there are no factors that can predict the treatment response to the next csDMARDs or biological DMARDs, this study suggested the potential of gut microbes as a predictor for treatment response.

Taken together, the current study shows that patients with established RA have a gut microbial composition distinct from that of healthy controls. Furthermore, gut microbes are potential biomarker candidates for predicting treatment responses in patients with established RA. The clinical significance and applicability of the gut microbiota in this context warrant further research and development.

## Supplementary Information

**Additional file 1: Supplemental figure 1. **Composition of gut microbiome from RA patients treated with bDMARD combined with or without csDMARDs. **A.** α-Diversity shown as Chao1, Shannon index, and Simpson's index. **B.** PCoA plot at the OTU level. **Supplemental figure 2. **Comparison of gut microbiome according to administration with or without methotrexate (MTX). **A.** α-Diversity shown as Chao1, Shannon index, and Simpson's index based on Bray-Curtis OTUs data. **B. **PCoA plot at the OTU level**. Supplemental figure 3. **Comparison of the gut microbiome according to response to all modified treatment strategies and the potential of gut microbes for predicting prognosis after the change of all modified treatment strategies therapy. The baseline gut microbial diversity and taxa in RA patients with moderate-to-high disease activity according to the response to all modified treatment strategies after 6 months. Responder was defined as patients whose ≤ 3.2 DAS28 at 6-month and non-responder was as those with > 3.2 DAS28 at 6-month. **A.** α-Diversity shown as Chao1, Shannon index, and Simpson's index. **B.** PCoA plot at the OTU level. **C.** LEfSe revealed specific microbes at genus level. **D.** The predictive potential of genera *Lanchnospiraceae *NK4A136 group and* Adlercreutizia *for predicting response to all modified therapy. ROC curve of each genus for predicting response to csDMARDs (left panel) and the area under the curve (AUC) and 95% confidence interval (right panel).

## Data Availability

The raw data of 16S rRNA amplicon sequences were deposited in Short Read Archive of NCBI (accession code PRJNA791216).

## Abbreviations

RA: Rheumatoid arthritis
ACPA: Anti-citrullinated protein antibody
DMARDs: Disease-modifying anti-rheumatic drugs
RF: Rheumatoid factor
DAS28: Disease Activity Score in 28 Joints
OTU: Operational taxonomic units
PCoA: Principal components analysis
RDA: Redundancy analysis
CCA: Canonical correspondence analysis
LDA: Linear discriminant analysis
LEfSe: LDA effect size
TNFi: Tumor necrosis factor α inhibitor

## References

[1] Smolen JS, Aletaha D, Barton A, Burmester GR, Emery P, Firestein GS, Kavanaugh A, McInnes IB, Solomon DH, Strand V (2018). Rheumatoid arthritis Nat Rev Dis Primers.

[2] Bergot AS, Giri R, Thomas R (2019). The microbiome and rheumatoid arthritis. Best Pract Res Clin Rheumatol.

[3] Scherer HU, Haupl T, Burmester GR (2020). The etiology of rheumatoid arthritis. J Autoimmun.

[4] Dedmon LE (2020). The genetics of rheumatoid arthritis. Rheumatology (Oxford).

[5] Kishikawa T, Maeda Y, Nii T, Motooka D, Matsumoto Y, Matsushita M, Matsuoka H, Yoshimura M, Kawada S, Teshigawara S (2020). Metagenome-wide association study of gut microbiome revealed novel aetiology of rheumatoid arthritis in the Japanese population. Ann Rheum Dis.

[6] Scher JU, Sczesnak A, Longman RS, Segata N, Ubeda C, Bielski C, Rostron T, Cerundolo V, Pamer EG, Abramson SB (2013). Expansion of intestinal Prevotella copri correlates with enhanced susceptibility to arthritis. Elife.

[7] Maeda Y, Kurakawa T, Umemoto E, Motooka D, Ito Y, Gotoh K, Hirota K, Matsushita M, Furuta Y, Narazaki M (2016). Dysbiosis contributes to arthritis development via activation of autoreactive T cells in the intestine. Arthritis Rheumatol.

[8] Alpizar-Rodriguez D, Lesker TR, Gronow A, Gilbert B, Raemy E, Lamacchia C, Gabay C, Finckh A, Strowig T (2019). Prevotella copri in individuals at risk for rheumatoid arthritis. Ann Rheum Dis.

[9] Wells PM, Adebayo AS, Bowyer RCE, Freidin MB, Finckh A, Strowig T, Lesker TR, Alpizar-Rodriguez D, Gilbert B, Kirkham B (2020). Associations between gut microbiota and genetic risk for rheumatoid arthritis in the absence of disease: a cross-sectional study. Lancet Rheumatol.

[10] Ivanov II, Atarashi K, Manel N, Brodie EL, Shima T, Karaoz U, Wei D, Goldfarb KC, Santee CA, Lynch SV (2009). Induction of intestinal Th17 cells by segmented filamentous bacteria. Cell.

[11] Rehaume LM, Mondot S, Aguirre de Cárcer D, Velasco J, Benham H, Hasnain SZ, Bowman J, Ruutu M, Hansbro PM, McGuckin MA (2014). ZAP-70 genotype disrupts the relationship between microbiota and host, leading to spondyloarthritis and ileitis in SKG mice. Arthritis Rheumatol.

[12] Abdollahi-Roodsaz S, Joosten LA, Koenders MI, Devesa I, Roelofs MF, Radstake TR, Heuvelmans-Jacobs M, Akira S, Nicklin MJ, Ribeiro-Dias F (2008). Stimulation of TLR2 and TLR4 differentially skews the balance of T cells in a mouse model of arthritis. J Clin Invest.

[13] Moutsopoulos NM, Kling HM, Angelov N, Jin W, Palmer RJ, Nares S, Osorio M, Wahl SM (2012). Porphyromonas gingivalis promotes Th17 inducing pathways in chronic periodontitis. J Autoimmun.

[14] Rooney CM, Mankia K, Emery P (2020). The role of the microbiome in driving RA-related autoimmunity. Front Cell Dev Biol.

[15] Cheng Z, Do T, Mankia K, Meade J, Hunt L, Clerehugh V, Speirs A, Tugnait A, Emery P, Devine D (2021). Dysbiosis in the oral microbiomes of anti-CCP positive individuals at risk of developing rheumatoid arthritis. Ann Rheum Dis.

[16] Maresz KJ, Hellvard A, Sroka A, Adamowicz K, Bielecka E, Koziel J, Gawron K, Mizgalska D, Marcinska KA, Benedyk M (2013). Porphyromonas gingivalis facilitates the development and progression of destructive arthritis through its unique bacterial peptidylarginine deiminase (PAD). PLoS Pathog.

[17] Kim JW, Jung H, Baek IP, Nam Y, Kang J, Chung MK, Park JB, Lee J, Kwok SK, Kim WU (2022). Differential effects of periodontal microbiome on the rheumatoid factor induction during rheumatoid arthritis pathogenesis. Sci Rep.

[18] Engström M, Eriksson K, Lee L, Hermansson M, Johansson A, Nicholas AP, Gerasimcik N, Lundberg K, Klareskog L, Catrina AI (2018). Increased citrullination and expression of peptidylarginine deiminases independently of P. gingivalis and A. actinomycetemcomitans in gingival tissue of patients with periodontitis. J Transl Med.

[19] Zhang X, Zhang D, Jia H, Feng Q, Wang D, Liang D, Wu X, Li J, Tang L, Li Y (2015). The oral and gut microbiomes are perturbed in rheumatoid arthritis and partly normalized after treatment. Nat Med.

[20] Zhou B, Xia X, Wang P, Chen S, Yu C, Huang R, Zhang R, Wang Y, Lu L, Yuan F (2018). Induction and amelioration of methotrexate-induced gastrointestinal toxicity are related to immune response and gut microbiota. EBioMedicine.

[21] Picchianti-Diamanti A, Panebianco C, Salemi S, Sorgi ML, Di Rosa R, Tropea A, Sgrulletti M, Salerno G, Terracciano F, D'Amelio R (2018). Analysis of gut microbiota in rheumatoid arthritis patients: disease-related dysbiosis and modifications induced by etanercept. Int J Mol Sci.

[22] Artacho A, Isaac S, Nayak R, Flor-Duro A, Alexander M, Koo I, Manasson J, Smith PB, Rosenthal P, Homsi Y (2021). The pretreatment gut microbiome is associated with lack of response to methotrexate in new-onset rheumatoid arthritis. Arthritis Rheumatol.

[23] Reyes-Castillo Z, Valdés-Miramontes E, Llamas-Covarrubias M, Muñoz-Valle JF (2021). Troublesome friends within us: the role of gut microbiota on rheumatoid arthritis etiopathogenesis and its clinical and therapeutic relevance. Clin Exp Med.

[24] Maeda Y, Takeda K (2019). Host-microbiota interactions in rheumatoid arthritis. Exp Mol Med.

[25] Kroese JM, Brandt BW, Buijs MJ, Crielaard W, Lobbezoo F, Loos BG, van Boheemen L, van Schaardenburg D, Zaura E, Volgenant CMC (2021). Differences in the oral microbiome in patients with early rheumatoid arthritis and individuals at risk of rheumatoid arthritis compared to healthy individuals. Arthritis Rheumatol.

[26] Aletaha D, Neogi T, Silman AJ, Funovits J, Felson DT, Bingham CO, Birnbaum NS, Burmester GR, Bykerk VP, Cohen MD (2010). 2010 Rheumatoid arthritis classification criteria: an American College of Rheumatology/European League Against Rheumatism collaborative initiative. Arthritis Rheum.

[27] Vrijhoef HJM, Diederiks JPM, Spreeuwenberg C, Van der Linden S (2003). Applying low disease activity criteria using the DAS28 to assess stability in patients with rheumatoid arthritis. Ann Rheum Dis.

[28] 16S metagenomic sequencing library preparation. [https://www.illumina.com/content/dam/illumina-support/documents/documentation/chemistry_documentation/16s/16s-metagenomic-library-prep-guide-15044223-b.pdf.]

[29] Herlemann DP, Labrenz M, Jurgens K, Bertilsson S, Waniek JJ, Andersson AF (2011). Transitions in bacterial communities along the 2000 km salinity gradient of the Baltic Sea. ISME J.

[30] Bolyen E, Rideout JR, Dillon MR, Bokulich NA, Abnet CC, Al-Ghalith GA, Alexander H, Alm EJ, Arumugam M, Asnicar F (2019). Reproducible, interactive, scalable and extensible microbiome data science using QIIME 2. Nat Biotechnol.

[31] Zakrzewski M, Proietti C, Ellis JJ, Hasan S, Brion MJ, Berger B, Krause L (2017). Calypso: a user-friendly web-server for mining and visualizing microbiome-environment interactions. Bioinformatics.

[32] Rantapää-Dahlqvist S, de Jong BA, Berglin E, Hallmans G, Wadell G, Stenlund H, Sundin U, van Venrooij WJ (2003). Antibodies against cyclic citrullinated peptide and IgA rheumatoid factor predict the development of rheumatoid arthritis. Arthritis Rheum.

[33] Breban M, Tap J, Leboime A, Said-Nahal R, Langella P, Chiocchia G, Furet JP, Sokol H (2017). Faecal microbiota study reveals specific dysbiosis in spondyloarthritis. Ann Rheum Dis.

[34] Chen J, Wright K, Davis JM, Jeraldo P, Marietta EV, Murray J, Nelson H, Matteson EL, Taneja V (2016). An expansion of rare lineage intestinal microbes characterizes rheumatoid arthritis. Genome Med.

[35] Sayers E, MacGregor A, Carding SR (2018). Drug-microbiota interactions and treatment response: Relevance to rheumatoid arthritis. AIMS Microbiol.

[36] El Menofy NG, Ramadan M, Abdelbary ER, Ibrahim HG, Azzam AI, Ghit MM, Ezz AS, Gazar YA, Salah M (2022). Bacterial compositional shifts of gut microbiomes in patients with rheumatoid arthritis in association with disease activity. Microorganisms.

[37] Peppercorn MA (1984). Sulfasalazine. Pharmacology, clinical use, toxicity, and related new drug development. Ann Intern Med.

[38] Audo R, Sanchez P, Rivière B, Mielle J, Tan J, Lukas C, Macia L, Morel J, Immediato Daien C. Rheumatoid arthritis is associated with increased gut permeability and bacterial translocation which are reversed by inflammation control. Rheumatology (Oxford). 2022:keac454. Online ahead of print. 10.1093/rheumatology/keac454 35946514

[39] Rafii F, Cerniglia CE (1995). Reduction of azo dyes and nitroaromatic compounds by bacterial enzymes from the human intestinal tract. Environ Health Perspect.

[40] Lee HJ, Zhang H, Orlovich DA, Fawcett JP (2012). The influence of probiotic treatment on sulfasalazine metabolism in rat. Xenobiotica.

[41] Van Hul M, Le Roy T, Prifti E, Dao MC, Paquot A, Zucker J-D, Delzenne NM, Muccioli GG, Clément K, Cani PD (2020). From correlation to causality: the case of Subdoligranulum. Gut Microbes.

[42] Alatawi H, Mosli M, Saadah OI, Annese V, Al-Hindi R, Alatawy M, et al. Attributes of intestinal microbiota composition and their correlation with clinical primary nonresponse to anti-TNF-α agents in inflammatory bowel disease patients. Bosn J Basic Med Sci 2022;22(3):412–26. 10.17305/bjbms.2021.6436 PMC9162754 34761733

[43] Nishiwaki H, Hamaguchi T, Ito M, Ishida T, Maeda T, Kashihara K, Tsuboi Y,  Ueyama J, Shimamura T, Mori H (2020). Short-chain fatty acid-producing gut microbiota is decreased in Parkinson’s disease but not in rapid-eye-movement sleep behavior disorder. mSystems.

[44] He J, Chu Y, Meng Q, Liu Y,  Jin J, Wang Y, Wang J, Huang B, Shi L (2022). Intestinal butyrate-metabolizing species contribute to autoantibody production and bone erosion in rheumatoid arthritis. Sci Adv.

[45] Crost EH, Tailford LE, Le Gall G, Fons M, Henrissat B, Juge N (2013). Utilisation of mucin glycans by the human gut symbiont Ruminococcus gnavus is strain-dependent. PLoS ONE.

[46] Henke MT, Kenny DJ, Cassilly CD, Vlamakis H, Xavier RJ, Clardy J (2019). Ruminococcus gnavus, a member of the human gut microbiome associated with Crohn’s disease, produces an inflammatory polysaccharide. Proc Natl Acad Sci U S A.

[47] Joensuu JT, Huoponen S, Aaltonen KJ, Konttinen YT, Nordström D, Blom M (2015). The cost-effectiveness of biologics for the treatment of rheumatoid arthritis: a systematic review. PLoS ONE.

[48] Yu D, Du J, Pu X, Zheng L, Chen S, Wang N, Li J, Chen S, Pan S, Shen B (2021). The gut microbiome and metabolites are altered and interrelated in patients with rheumatoid arthritis. Front Cell Infect Microbiol.

[49] Wu X, Liu J, Xiao L, Lu A, Zhang G (2017). Alterations of gut microbiome in rheumatoid arthritis. Osteoarthr Cartil.

[50] Jin M, Kalainy S, Baskota N, Chiang D, Deehan EC, McDougall C, Tandon P, Martínez I, Cervera C, Walter J (2019). Faecal microbiota from patients with cirrhosis has a low capacity to ferment non-digestible carbohydrates into short-chain fatty acids. Liver Int.

[51] Martinsson K, Dürholz K, Schett G, Zaiss MM, Kastbom A (2022). Higher serum levels of short-chain fatty acids are associated with non-progression to arthritis in individuals at increased risk of RA. Ann Rheum Dis.

[52] Takeshita K, Mizuno S, Mikami Y, Sujino T, Saigusa K, Matsuoka K, Naganuma M, Sato T, Takada T, Tsuji H (2016). A single species of Clostridium subcluster XIVa decreased in ulcerative colitis patients. Inflamm Bowel Dis.

