# Supplementary material for: Factors associated with the composition of the gut microbiome in patients with established rheumatoid arthritis and its value for predicting treatment responses
Source: Arthritis Res Ther. 2023 Mar 2;25:32. doi: 10.1186/s13075-023-03013-x (PMC9979421; doi:10.1186/s13075-023-03013-x)
Supplement: Supplementary file 1 — Additional file 1: Supplemental figure 1. Composition of gut microbiome from RA patients treated with bDMARD combined with or without csDMARDs. A. α-Diversity shown as Chao1, Shannon index, and Simpson's index. B. PCoA plot at the OTU level. Supplemental figure 2. Comparison of gut microbiome according to administration with or without methotrexate (MTX). A. α-Diversity shown as Chao1, Shannon index, and Simpson's index based on Bray-Curtis OTUs data. B. PCoA plot at the OTU level. Supplemental figure 3. Comparison of the gut microbiome according to response to all modified treatment strategies and the potential of gut microbes for predicting prognosis after the change of all modified treatment strategies therapy. The baseline gut microbial diversity and taxa in RA patients with moderate-to-high disease activity according to the response to all modified treatment strategies after 6 months. Responder was defined as patients whose ≤ 3.2 DAS28 at 6-month and non-responder was as those with > 3.2 DAS28 at 6-month. A. α-Diversity shown as Chao1, Shannon index, and Simpson's index. B. PCoA plot at the OTU level. C. LEfSe revealed specific microbes at genus level. D. The predictive potential of genera Lanchnospiraceae NK4A136 group and Adlercreutizia for predicting response to all modified therapy. ROC curve of each genus for predicting response to csDMARDs (left panel) and the area under the curve (AUC) and 95% confidence interval (right panel). [file 13075_2023_3013_MOESM1_ESM.docx]

**Supplemental Information**

**The gut microbiome in patients with established rheumatoid arthritis:** factors associated with composition, and its value for predicting treatment responses

Jung Hee Koh^1,2,a^, Eun Ha Lee^3,a^, Kwang Hyun Cha^3^, Cheol-Ho Pan^3^, Donghyun Kim^4.5.6^, and Wan-Uk Kim^1,2^

^1^Division of Rheumatology, Department of Internal Medicine, the Catholic University of Korea, School of Medicine, Seoul 06591, Republic of Korea

^2^ Center for Integrative Rheumatoid Transcriptomics and Dynamics, School of Medicine, the Catholic University of Korea, Seoul 06591, Republic of Korea

^3^Natural Product Informatics Research Center, KIST Gangneung Institute of Natural Products, Gangneung 25451, Korea

^4^Department of Biomedical Sciences, Seoul National University College of Medicine, Seoul, Republic of Korea

^5^Department of Microbiology and Immunology, Seoul National University College of Medicine, Seoul, Republic of Korea

^6^Institute of Endemic Diseases, Seoul National University Medical Research Center, Seoul, Republic of Korea

^a^ These authors contributed equally to this work.

*Correspondence and reprint requests to:

Dr. Wan-Uk Kim,

Division of Rheumatology, Department of Internal Medicine, The Catholic University of Korea, School of Medicine, Seoul, Republic of Korea (E-mail: [wan725@catholic.ac.kr](mailto:wan725@catholic.ac.kr))

Or Dr. Donghyun Kim,

Department of Microbiology and Immunology, Seoul National University College of Medicine, Seoul, Republic of Korea (E-mail: [biologokim@snu.ac.kr](mailto:biologokim@snu.ac.kr))


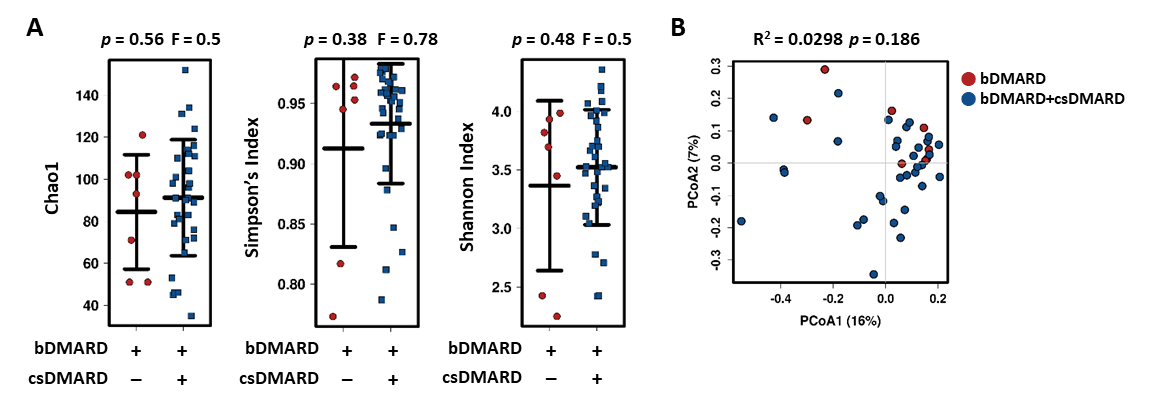


**Supplemental figure 1.** Composition of gut microbiome from RA patients treated with bDMARD combined with or without csDMARDs. **A.** α-Diversity shown as Chao1, Shannon index, and Simpson's index. **B.** PCoA plot at the OTU level.


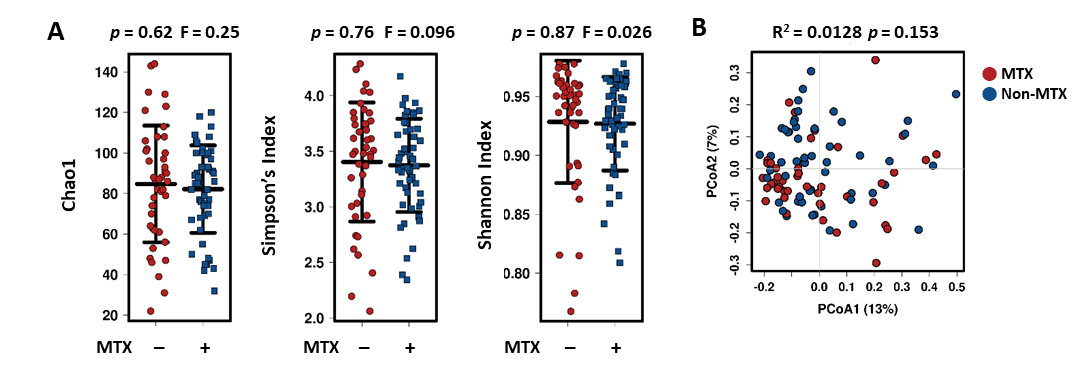


**Supplemental figure 2.** Comparison of gut microbiome according to administration with or without methotrexate (MTX). **A.** α-Diversity shown as Chao1, Shannon index, and Simpson's index based on Bray-Curtis OTUs data. **B.** PCoA plot at the OTU level**.**


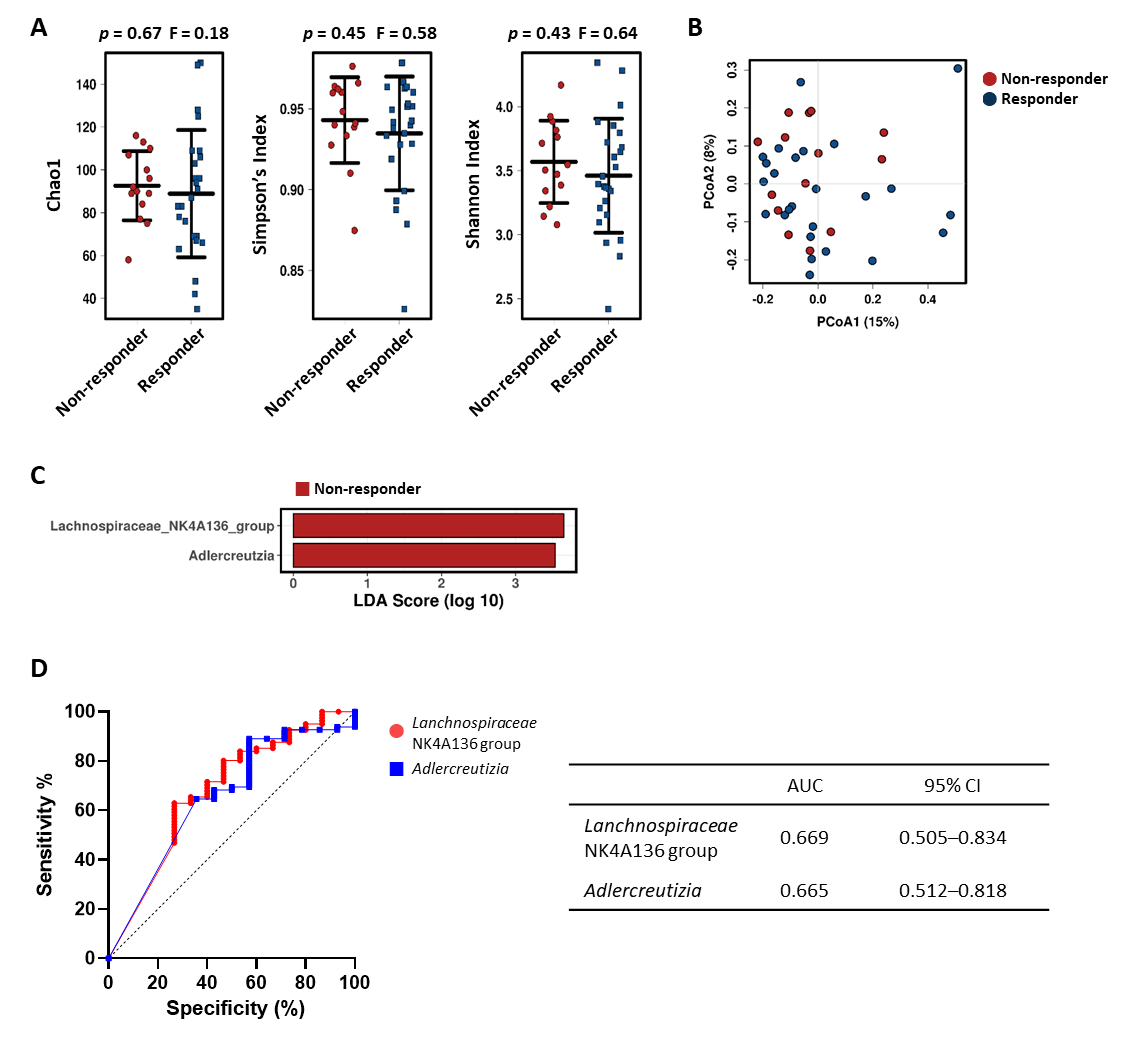


**Supplemental figure 3.** Comparison of the gut microbiome according to response to all modified treatment strategies and the potential of gut microbes for predicting prognosis after the change of all modified treatment strategies therapy.

The baseline gut microbial diversity and taxa in RA patients with moderate-to-high disease activity according to the response to all modified treatment strategies after 6 months. Responder was defined as patients whose ≤ 3.2 DAS28 at 6-month and non-responder was as those with > 3.2 DAS28 at 6-month. **A.** α-Diversity shown as Chao1, Shannon index, and Simpson's index. **B.** PCoA plot at the OTU level. **C.** LEfSe revealed specific microbes at genus level. **D.** The predictive potential of genera *Lanchnospiraceae* NK4A136 group and *Adlercreutizia* for predicting response to all modified therapy. ROC curve of each genus for predicting response to csDMARDs (left panel) and the area under the curve (AUC) and 95% confidence interval (right panel).
